# Initial Trials With Susceptibility-Based and Empiric Anti-*H. pylori* Therapies in Mongolia

**DOI:** 10.3389/fphar.2019.00394

**Published:** 2019-04-16

**Authors:** Tsogt-Ochir Byambajav, Namdag Bira, Gotov Choijamts, Duger Davaadorj, Boldbaatar Gantuya, Tserenchimed Sarantuya, Gidaagaya Sarantuya, Altangerel Enkhtsetseg, Dungubat Erdenetsogt, Adiyasuren Battulga, Tegshee Tserentogtokh, Takeshi Matsuhisa, Yoshio Yamaoka, Khasag Oyuntsetseg

**Affiliations:** ^1^Department of Gastroenterology and Hepatology, School of Medicine, Mongolian National University of Medical Sciences, Ulaanbaatar, Mongolia; ^2^Department of Pharmacology, Otoch Manramba University of Mongolia, Ulaanbaatar, Mongolia; ^3^Department of Environmental and Preventive Medicine, Faculty of Medicine, Oita University, Yufu, Japan; ^4^Department of Internal Medicine, Intermed Hospital, Ulaanbaatar, Mongolia; ^5^Department of Laboratory, General Hospital of Defense and Law Enforcement, Ulaanbaatar, Mongolia; ^6^Department of Endoscopy, Ulaanbaatar Songdo Hospital, Ulaanbaatar, Mongolia; ^7^Department of Gastroenterology, State Central Third Hospital, Ulaanbaatar, Mongolia; ^8^Department of Gastroenterology, Nippon Medical School, Tama-Nagayama University Hospital, Tama-Nagayama, Japan; ^9^Gastroenterology and Hepatology Section, Department of Medicine, Baylor College of Medicine, Houston, TX, United States

**Keywords:** *Helicobacter pylori*, antibiotic resistance, eradication therapy, empirical therapy, Mongolia

## Abstract

**Background:** Mongolia has a high prevalence of *Helicobacter pylori* infection and gastric cancer. We conducted a prospective, randomized, single-blind study to evaluate the efficacy of common regimens in Mongolia and to obtain specimens for susceptibility testing.

**Methods:** Empiric treatments: 270 patients with confirmed *H. pylori* infection were randomized to receive 10 days clarithromycin-triple therapy (Clari-TT) (*n* = 90), modified bismuth quadruple therapy (M-BQT) (*n* = 90), or sequential therapy (ST) (*n* = 90). A second group of 46 patients received susceptibility-based Clari-TT. *H. pylori* was cultured from 131 patients and susceptibility testing was performed. *H. pylori* eradication was confirmed by stool antigen 4 weeks after the therapy.

**Results:** Intention-to-treat (ITT) analysis cure rates were 71.1% (95% CI = 61.7–80.5%) for Clari-TT, 87.8% (95% CI = 81–94.6%) for M-BQT, 67.8% (95% CI = 58.1–77.5%) for ST vs. 89.1% (95% CI = 86–98.2%) for susceptibility-based Clari-TT. Per-protocol (PP) analysis results for these therapies were 72.7% (63.4–82%), 89.8% (83.5–96.1%), 68.5% (58.8–78.2%), and 97.6% (89.5–99.8%), respectively. Among 131 cultured *H. pylori*, resistance rates to amoxicillin, clarithromycin, and metronidazole were 8.4, 37.4, and 74%, respectively.

**Conclusion:** In Mongolia, the prevalence of *H. pylori* resistance is high requiring bismuth quadruple therapy or susceptibility-based therapy to obtain acceptable cure rates.

## Introduction

As in most developing countries, the prevalence of *Helicobacter pylori* infection is high in Mongolia (Nyamdavaa, 2013) with reported prevalence ranging of 80% among adults (Matsuhisa et al., 2015; Khasag et al., 2018), 64% among adolescents, and 65 and 100% among pediatric patients with gastric comorbidity (Go, 2013). Gastric cancer is a common problem in Mongolia; an age-standardized rate of 33.1 per 100,000, which is the second highest incidence in the world (International Agency for Research on Cancer; GLOBOCAN2018). Information regarding *H. pylori* antibiotic resistance in Mongolia is scanty with a single prior study reporting the resistance rates of 35.5% for clarithromycin, 68.4% for metronidazole, 23% for amoxicillin, 25% for tetracycline, 26.2% for erythromycin, and 14.5% for nitrofurantoin (Bolor-Erdene et al., 2017). Here, we assessed the primary resistance of *H. pylori* strains isolated from adults and compared antimicrobial treatment efficacy of commonly used regimens in Mongolian patients with dyspepsia.

## Materials and Methods

### Patients, *H. pylori* Detection Tests, Treatment, and Randomization

This study was done between November 2013 and March 2017 in Ulaanbaatar, the capital city of Mongolia where more than half of the population of Mongolia lives. Study population consists of two groups. The initial study was to examine commonly used empiric therapies. The study was done at the Department of Endoscopy at University Hospital of Mongolian National University of Medical Sciences between March 2014 and March 2017 and consisted of 270 patients with documented *H. pylori* infections (42 women and 89 men; mean age 37 years; range 17–79 years). Using a computer-generated number sequence, eligible *H. pylori*-infected patients were randomly assigned to the Clari-TT group (*n* = 90): 40 mg pantoprazole (KRKA.dd. Novo Mesto, Slovenia) twice daily, 1 g amoxicillin (Astellas Pharma Europe BV, Netherlands) twice daily, and 500 mg clarithromycin (Fromilid KRKA.dd. Novo Mesto, Slovenia) twice daily for 10 days, a M-BQT containing amoxicillin and clarithromycin-containing group (*n* = 90): 40 mg pantoprazole twice daily, 240 mg bismuth tripotassium dicitrate (Astellas Pharma Europe BV, Netherlands) twice daily, 1 g amoxicillin twice daily, and 500 mg clarithromycin twice daily for 10 days. The final group received 10-day ST group (*n* = 90) consisting of 40 mg pantoprazole twice daily and 1 g amoxicillin twice daily for 5 days, followed by 40 mg pantoprazole twice daily, 500 mg clarithromycin twice daily, and 500mg metronidazole (PT. Otto Pharmaceutical Industries) twice daily for 5 additional days (Table 1). An independent research assistant generated the computerized random number sequence. Before enrollment, the status of *H. pylori* infection was confirmed by a locally developed rapid urease test (Mon HP test, developed at Mongolian National University of Medical Sciences), histology with hematoxylin and eosin and modified Giemsa staining, and HpStAg test (SD. *H. pylori* Ag ELISA kit, Korea). Patients with positive results in at least two of these tests were eligible for enrollment.

**Table 1:** *Helicobacter pylori* eradication therapies.

	Regimens	Drugs	Dose	Daily	Days
Empirical therapies	Clari-TT^a^ (*n* = 90)	Pantoprazole^∗^ Amoxicillin Clarithromycin	40 mg 1 g 500 mg	Twice Twice Twice	10 10 10
	M-BQT^b^ (*n* = 90)	Pantoprazole^∗^ Bismuth tripotassium dicitrate Amoxicillin Clarithromycin	40 mg 240 mg 1 g 500 mg	Twice Twice Twice Twice	10 10 10 10
	ST^c^ (*n* = 90)	Pantoprazole^∗^ Amoxicillin Followed by Pantoprazole Clarithromycin Metronidazole	40 mg 1 g 40 mg 500 mg 500 mg	Twice Twice Twice Twice Twice	5 5 5 5 5
Susceptibility-based Clari-TT (*n* = 46)		Pantoprazole^∗^ Amoxicillin Clarithromycin	40 mg 1 g 500 mg	Twice Twice Twice	10 10 10

*^∗^An additional 2-week monotherapy with 40 mg pantoprazole once daily 7 days, followed by 20 mg pantoprazole once daily 7 days following eradication therapy. ^*a*^Clarithromycin-triple therapy, ^*b*^modified bismuth quadruple therapy, ^*c*^sequential therapy.*

A second experiment was a multicenter study done between November 2013 and June 2014 in Ulaanbaatar and consisted of 225 patients with dyspepsia who visited endoscopy departments of one of three centers in Ulaanbaatar; University Hospital of Mongolian National University of Medical Sciences, Ulaanbaatar Songdo Hospital, and The Third State Central Hospital. All underwent gastroscopy with multiple biopsies for rapid urease test, histology and *H. pylori* culturing. *H. pylori* strains were isolated from 131 patients and further tested for antibiotic resistance. Metronidazole-based triple therapy was received the patients with strains resistant to clarithromycin (*n* = 9). If the *H. pylori*-isolates were resistance to metronidazole and clarithromycin, but sensitive to amoxycillin (*n* = 21) levofloxacin-based triple therapy. These data were excluded from the study because of few patient number (metronidazole-based triple therapy) and lacking the susceptibility test of levofloxacin (levofloxacin-based triple therapy). Forty-six patients (29 women and 17 men; mean age 39 years; range 24–54 years) were infected with *H. pylori* susceptible to amoxicillin and clarithromycin and were treated with susceptibility-based Clari-TT (*n* = 46) consisting of 40 mg pantoprazole twice daily, 1 g amoxicillin twice daily, and 500 mg clarithromycin twice daily for 10 days (Table 1). PPIs were given a half-hour before breakfast and the evening meal while antibiotics were given following these meals. In each group, an additional 2-week monotherapy with 40 mg pantoprazole once daily 7 days, followed by 20 mg pantoprazole once daily 7 days following eradication therapy. Cure rates were assessed using HpStAg test 28 days after the termination of treatment.

Exclusion criteria were: age < 18 years; previous *H. pylori* eradication therapy; consumption of PPI, histamine H_2_-receptor antagonists, bismuth and/or antibiotics, concomitant anticoagulant, non-steroid anti-inflammatory drugs, or ketoconazole within the previous 4 weeks; patients with allergic history to the medications used; previous surgery of the stomach, including endoscopic mucosal or submucosal resection for adenoma or early gastric cancer; patients with peptic ulcer diseases and gastric cancer; the coexistence of serious concomitant illness (e.g., decompensated liver cirrhosis or kidney failure); alcohol abuse; pregnancy or lactation; Zollinger–Ellison syndrome; hematological disorders; and severe psychiatric or neurological disorders.

### Antibiotic Susceptibility Testing

Experienced endoscopists collected gastric biopsy specimens during each endoscopy session. Biopsy specimens for culture were immediately placed in transport media containing 20% glycerol at -20°C, and stored at -80°C within a day of collection until used for culture testing. For *H. pylori* culture, one antral biopsy specimen was homogenized in saline and inoculated onto Mueller Hinton II Agar medium (Becton Dickinson, Franklin Lakes, NJ, United States) supplemented with 7% horse blood without antibiotics. The plates were incubated for up to 14 days at 37°C under microaerophilic conditions (10% O_2_, 5% CO_2_, and 85% N_2_). *H. pylori* isolates were identified on the basis of colony morphology, Gram staining results, and positive reactions for oxidase, catalase, and urease. Isolated strains were stored at -80°C in Brucella Broth (Difco, Franklin Lakes, NJ, United States) containing 10% dimethyl sulfoxide and 10% horse serum.

The serial twofold agar dilution method was used to determine the MIC of amoxicillin (Sigma Chemical Co., St Louis, MO, United States), clarithromycin (Abbott Laboratories, Abbott Park, IL, United States), and metronidazole (Sigma). Briefly, bacteria were subcultured on Mueller Hinton II Agar medium (Becton Dickinson) supplemented with 10% defibrinated horse blood. The bacterial suspension, adjusted to be equivalent to a McFarland opacity standard of 3.0, was used to inoculate each plate. After 72 h incubation, the MIC of each antibiotic was determined. Quality control was performed using *H. pylori* ATCC 43504. The resistance breakpoints were determined by the European Committee on Antimicrobial Susceptibility Testing (EUCAST; available at www.eucast.org). Strains were considered as resistant when the MIC was more than 0.125 mg/L for amoxicillin, >0.25 mg/L for clarithromycin, and >8 mg/L for metronidazole.

### Adverse Events and Compliance

At the end of the treatment, both side effects and therapeutic compliance were assessed by personal interview. The patients were informed of the common adverse events of drugs before treatment initiation and were asked to record the symptoms during treatment in provided diaries. Adverse events were assessed according to a four-point scale system: 1 = none, 2 = mild (discomfort annoying but not interfering with daily life), 3 = moderate (discomfort sufficient to interfere with daily life), and 4 = severe (discomfort resulting in discontinuation of eradication therapy). Compliance to treatment was considered excellent if the patient took more than 90% of the medication, moderate if the patient took 70–90% of the medication, and poor if the patient took less than 70% of medications.

### Statistical Analysis

All statistical analyses were performed using the SPSS software (ver. 20.0, SPSS Inc., Chicago, IL, United States). Categorical variables are reported as numbers and percentages and compared using the chi-square test or Fisher’s exact test. Continuous variables are reported as means ± SD and were compared using *t-*tests. A multivariate logistic regression model including age and sex was used to calculate the OR of the resistance places. The OR and 95% CI were used to estimate the risk. Comparisons between patient groups were performed by using the *t*-test for unpaired data, the Chi-squared test and Tukey test as appropriate. The eradication rates with their 95% CI were calculated at both “ITT” and at “PP” analyses. At ITT, all the enrolled patients were included, while at PP only compliant patients who had done HpStAg test control were considered. Two-tailed *p*-values of less than 0.05 were considered statistically significant.

### Ethics Statement

All procedures contributing to this work complied with the ethical standards of the relevant national and institutional committees on human experimentation and with the Helsinki Declaration of 1975, as revised in 2008. The study protocol was approved by the Institutional Ethical Committee of Mongolian National University of Medical sciences (Ulaanbaatar, Mongolia) and Oita University Faculty of Medicine (Yufu, Japan). Written informed consent was obtained from all participants prior to testing and prior to treatment.

## Results

### Result of the Empirical Therapies

**FIGURE 1:**
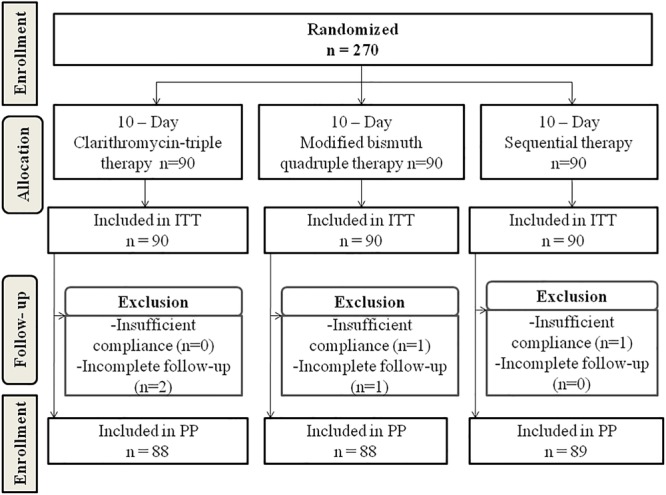
Flowchart of the patients in the study.

A total of 270 *H. pylori*-infected patients were randomly assigned to receive Clari-TT (*n* = 90), M-BQT (*n* = 90), and ST (*n* = 90). The data regarding the clinical characteristics of the patients are summarized in Table 2. All subjects treated were included in the ITT analysis for *H. pylori* eradication. A total of 265 patients completed the study. Two patients in the Clari-TT group and one patient in the M-BQT group did not complete follow-up HpStAg ELISA testing. One patient in the M-BQT group and one patient in the ST group interrupted therapy because of severe adverse effects. Therefore, the Clari-TT, M-BQT, and ST groups had 88, 88, and 89 patients for PP analysis, respectively (Figure 1). Compliance to treatment was excellent 98, 98, and 99% in Clari-TT, M-BQT, and ST groups, respectively.

**Table 2:** Demographic and clinical characteristics of the enrolled patients with the *Helicobacter pylori* eradication therapies.

Characteristic	Susceptibility-based Clari-TT^a^	Empirical therapies			
		Clari-TT^a^	M-BQT^b^	ST^c^	*p*-Value
Total patients	46	90	90	90	–
Age (years)	39 ± 15^∗^	38.7 ± 15.6^∗^	37 ± 11.8^∗^	37.1 ± 12.7^∗^	0.64
Sex (male/female)	17/29	28/62	42/48	39/51	0.08
Smoking habit (yes/no)	9/36	11/79	14/76	22/68	0.82
Alcohol consumption (yes/no or less than standard drink)	8/38	12/78	11/79	17/73	0.68

*^∗^mean ± SD, ^*a*^Clarithromycin-triple therapy, ^*b*^modified bismuth quadruple therapy, ^*c*^sequential therapy.*

According to ITT analysis, cure rates for empiric therapies were 71.1% (95% CI = 61.7–80.5%) for Clari-TT, 87.8% (95% CI = 81–94.6%) for M-BQT, and 67.8% (95% CI = 58.1–77.5%) for ST; PP analysis results for these therapy were 72.7% (95% CI = 63.4–82%), 89.8% (95% CI = 83.5–96.1%), and 68.5% (95% CI = 58.8–78.2%), respectively (Table 3). Only M-BQT achieved acceptable or near acceptable cure rates (i.e., 87.8% ITT and 89.8% PP) (Table 3 and Figure 3).

**Table 3:** *Helicobacter pylori* eradication rates by ITT analysis and PP analysis fist-line therapies.

Therapy	Susceptibility-based Clari-TT^a^ % (*n*)	Empirical therapies			*P*-value
		Clari-TT %(*n*)	M-BQT^b^ %(*n*)	ST^c^ %(*n*)	
ITT analysis		
*H. pylori* cure rate	89.1% (41/46)	71.1% (64/90)	87.8% (79/90)	67.8% (61/90)	<0.0001
95%CI	86–98.2	61.7–80.5	81–94.6	58.1–77.5	
*P*-value^∗^		
Clari-TT^a^	0.021	–	0.071	0.988	
M-BQT^b^	0.947	–		0.03	
ST^c^	0.008	–	–	–	
PP analysis		
*H. pylori* eradication rate	97.6% (41/42)	72.7% (64/88)	89.8% (79/88)	68.5% (61/89)	<0.0001
95%CI	89.5–99.8	63.4–82	83.5–96.1	58.8–78.2	
*P*-value^∗^		
Clari-TT^a^	0.002	–	0.017	0.884	
M-BQT^b^	0.853	–	–	0.001	
ST^c^	<0.0001	–	–	–	

*^∗^*P*-values from pairwise comparison made by Tukey’s all-pairwise test. ^*a*^Clarithromycin-triple therapy, ^*b*^modified bismuth quadruple therapy, ^*c*^sequential therapy.*

Overall 12.5, 4.5, and 22.2% patients in the Clari-TT, M-BQT, and ST groups complained of side effects (*p* = 0.02). The nausea and headache were more commonly presented side effects in ST than the Clari-TT and M-BQT (*p* = 0.0001) (Table 4).

**Table 4:** Adverse effects reported by the patients during *Helicobacter pylori* eradication therapies.

Adverse event	Susceptibility-based Clari-TT^a^	Empirical therapies			*P*-value
		Clari-TT	M-BQT^b^	ST^c^	
	Total number of patients			
	(Number of patients with			
	mild/moderate/severe adverse events)				
Nausea	2(0/2/0)	3(2/1/0)	0(0/0/0)	22(12/10/0)	0.0001
Vomiting	0(0/0/0)	0(0/0/0)	0(0/0/0)	1(1/0/0)	0.38
Taste perversion	1(1/0/0)	1(1/0/0)	2(1/0/1)	0(0/0/0)	0.36
Abdominal pain	1(1/0/0)	4(2/2/0)	1(1/0/0)	5(2/3/0)	0.27
Diarrhea	1(1/0/0)	5(3/2/0)	2(2/0/0)	1(1/0/0)	0.18
Headache	0(0/0/0)	2(2/0/0)	1(1/0/0)	12(6/5/1)	0.0001
Skin rash	4(4/0/0)	3(2/1/0)	0(0/0/0)	3(3/0/0)	0.23
Candida	2(2/0/0)	3(3/0/0)	1(1/0/0)	3(3/0/0)	0.56
Others	2(0/2/0)	0(0/0/0)	1(1/0/0)	1(1/0/0)	0.23
Total	9.5%(4/42)	12.5(11/88)	4.5%(4/89)	22.2%(20/90)	0.02

*^*a*^Clarithromycin-triple therapy, ^*b*^modified bismuth quadruple therapy, ^*c*^sequential therapy.*

### Antimicrobial Susceptibility/Resistance

**FIGURE 2:**
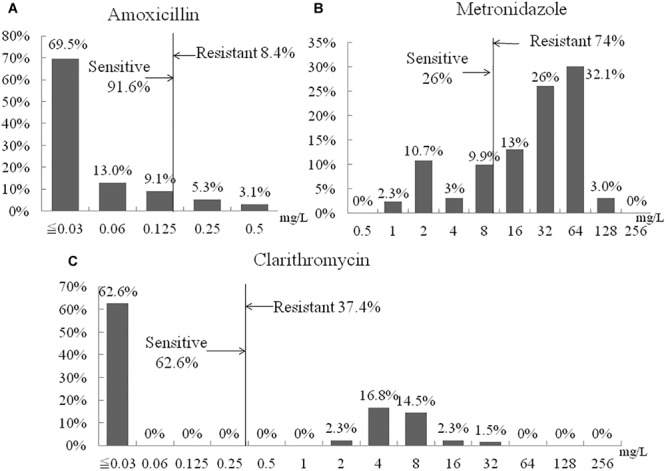
Distribution of antibiotic MIC values. **(A**) Amoxicillin. **(B)** Metronidazole. **(C)** Clarithromycin.

Susceptibility testing showed that overall resistance to all tested antibiotics was high [e.g., 11 (8.4%) resistant to amoxicillin, 49 (37.4%) resistant to clarithromycin, and 97 (74%) to metronidazole] (Figure 2). Only 20 (15.3%) were susceptible to all three antibiotics and 40 (30.5%) were resistant to at least two antibiotics. One (0.8%) isolate showed resistance to amoxicillin and clarithromycin and six (4.8%) were R, to all three antibiotics (Table 5). The proportion of strains resistant to metronidazole was lower in the aged <29 years and >60 years groups compared to the other age groups (*p* = 0.03). Resistances to the other two antibiotics and multidrug resistance were independent of age (Table 6).

**Table 5:** Multidrug resistance.

*Helicobacter pylori* Antimicrobial susceptibility testing				S or R to multidrug
Amoxicillin	Clarithromycin	Metronidazole	*N* (%)	
S^a^	S	S	20 (15.3)	S to multidrug 91 (69.5%)
S	S	R^b^	58 (44.3)	
S	R	S	11 (8.4)	
R	S	S	2 (1.5)	
R	R	R	6 (4.8)	R to multidrug 40 (30.5%)
R	R	S	1 (0.8)	
R	S	R	2 (1.5)	
S	R	R	31 (23.7)	

*^*a*^S-S, ^*b*^R-R.*

**Table 6:** Relationship between age and antibiotic resistance of *Helicobacter pylori*.

Antimicrobial susceptibility testing result	Age groups					
	≤29(38) *n* (%)	30–39(35) *n* (%)	40–49(25) *n* (%)	50–59(17) *n* (%)	≥60 (16) *n* (%)	*P*-value
R to multiple drugs (40)	6 (15.8)	14 (40)	10 (40)	4 (23.5)	6 (37.6)	0.126
Metronidazole-R^a^ (97)	21 (55.3)	29 (82.9)	21 (84)	16 (94.1)	10 (62.5)	0.03
Amoxicillin-R (11)	5 (13.2)	1 (2.9)	3 (12)	1 (5.9)	1 (6.3)	0.53
Clarithromycin-R (49)	11 (28.9)	14 (40)	12 (48)	4 (23.5)	8 (50)	0.29

*^*a*^R – R.*

### Result of the Susceptibility-Based Therapy

**FIGURE 3:**
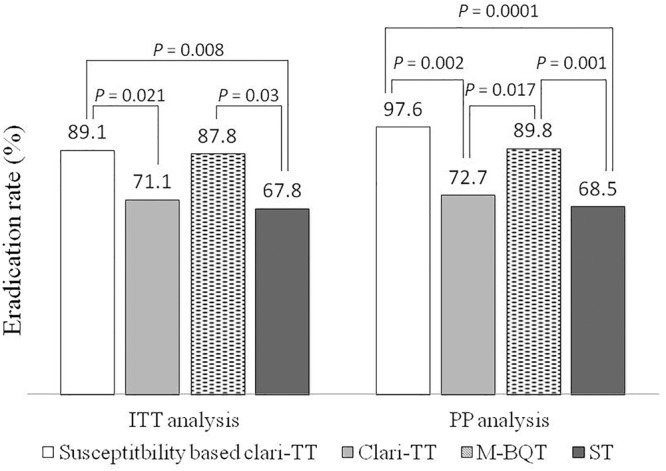
Helicobacter pylori eradication rates of the treatment groups according to the ITT and PP analysis. ITT – intention-to-treat; PP – per protocol; Clar-TT – clarithromycin-triple therapy; M-BQT – modified bismuth quadruple therapy; ST – sequential therapy.

Forty-six patients (29 women and 17 men; mean age 39 years; range 24–54 years) received susceptibility-based Clari-TT. Although 78 strains were sensitive for both amoxicillin and clarithromycin, 32 subjects refused to participate. Among 46 patients enrolled, 37 (80.3%) were non-smokers and 38 (81.6%) either did not consume alcohol or took less than standard drink/day (Table 2). All enrolled were included in the ITT analysis. A total of 42 patients completed susceptibility-based Clari-TT and four did not receive a confirmation of cure HpStAg ELISA test. The cure rates were: ITT 89.1% (95% CI = 86–98.2%) and PP 97.6% (95% CI = 89.5–99.8%), respectively (Table 3). Total of 9.5% patients in the susceptibility-based Clari-TT regimen had side effects (Table 4). The cure rates for susceptibility-based Clari-TT were significantly greater than those when the same regime was used as an empiric therapy (Table 3 and Figure 3).

## Discussion

*Helicobacter pylori* infection is an infectious disease and it has been suggested that ideal regimens should be able to cure more than 95% of cases adherent to the regimen. According to one classification (Graham et al., 2007), the efficacy of *H. pylori* eradication regimens is considered as: A = excellent (>95% PP eradication rate), B = good (90–95%), C = fair (85–89%), D = poor (81–84%), and F = unacceptable (≤80% PP eradication rate). In Mongolia, empiric therapy with Clari-TT and ST regimens was unacceptable as empiric therapies. However, susceptibility-based therapy and empiric M-BQT were classified as excellent and good regimens with PP cure rates of 98 and 90%, respectively.

In Mongolia, there are no previous data *H. pylori* cure rates. The high prevalence of clarithromycin and metronidazole resistance in Mongolia undermined the efficacy of empiric Clari-TT and ST (Rossum, 1999; Janssen et al., 2001; Horvath et al., 2012; Kate et al., 2013; Yoon et al., 2013; Zullo et al., 2013). *H. pylori* resistance to metronidazole was very high (74%) in Mongolia which is consistent with other studies from developing countries, possibly owing to the common use of metronidazole to treat parasitic infections, periodontal, and gynecological diseases in developing countries. Among other Asia-Pacific countries, China, Vietnam, Bhutan, and Bangladesh and Nepal had the highest prevalence of metronidazole resistance (61, 72, 83, 84, and 88%, respectively) (Kuo et al., 2017). Loss of penicillin-binding protein is known to be associated with amoxicillin resistance (Gerrits et al., 2002). *H. pylori* resistance to amoxicillin in Mongolia was higher (8.4%) than in most other countries (Horiki et al., 2009; Caliskan et al., 2015; Miftahussurur and Yamaoka, 2015; Boyanova et al., 2016). However, increasing amoxicillin primary resistance rates have been reported in South Korea (7.1–18.5%) (Lee et al., 2013). In our previous study (Bolor-Erdene et al., 2017), we had also reported very high rate of amoxicillin resistance. We think several possibilities as follows; poor compliances of drug regulation policy (i.e., no prescription is required) and amoxicillin is one of the most commonly prescribed antibiotics for patients suffer from respiratory tract, ear, nose, and throat infection, which is a leading cause of population morbidity with 1579.9 cases per 10,000 population (Health indicators of Mongolia for WPRO, WHO database. [Internet]. 2017. Available from: http://www.chd.mohs.mn). Amoxicillin is the first-line treatment for these infections, and combination use of clarithromycin has increased recently.

The Maastricht (Malfertheiner et al., 2016) and Toronto Consensus (Fallone et al., 2016) recommend 14-day bismuth, tetracycline, metronidazole, PPI quadruple therapy M-BQT, or susceptibility-based therapy be used in regions with high clarithromycin resistance. The results from this study provided evidence that our modified bismuth quadruple and susceptibility-based therapies were more effective than Clari-TT and ST in a country with high resistance for clarithromycin and metronidazole. BQT is a 20-year-old regimen that consists of PPIs plus bismuth salt, tetracycline, and metronidazole (Der Hulst et al., 1996). We used a M-BQT due to unavailability of tetracycline in Mongolia. Recently, Dore et al. (2016) reported that addition of bismuth to Clari-TT increase treatment efficacy by 30–40%. The mechanisms of bismuth in the eradication of *H. pylori* remain unclear. The most recent *in vitro* study reported that bismuth impeded proton entry into the organisms potentially impairing their ability to respond to acid and enhancing the efficacy of growth-dependent antibiotics (Marcus et al., 2015). Recent studies have suggested that tetracycline can be replaced by amoxicillin 1 g t.i.d. with excellent results in highly resistant populations (Chen et al., 2016; Graham et al., 2018a).

The limitations of this study include the fact that it conducted on a small sample size, taken from a geographically limited population in Ulaanbaatar, Mongolia. Other limitations included use of low dose PPI therapy (i.e., pantoprazole 40 mg is equivalent to 9 mg of omeprazole) (Graham et al., 2018b). Recent recommendations recommend double-dose PPI which is equivalent to at least 40 mg of omeprazole/dose. All recent recommendations also recommend that the duration of therapy be 14 days (Malfertheiner et al., 2016; Graham et al., 2018a). Subsequent studies to compare 10 and 14 day therapies are needed. In addition, in Japan, the dosage of clarithromycin is lower (i.e., 200 in stead of 500 mg/dose) (Lee, 2014) and studies using this lower dose in Mongolia might also result in improved compliance due to reduced side effects.

## Conclusion

*Helicobacter pylori* resistance rate to metronidazole, clarithromycin, and amoxicillin are high in Mongolia. These initial studies have shown that highly successful therapy is possible using empiric bismuth quadruple therapies and susceptibility-based therapy. Subsequent studies are needed to identify the optimum drugs, doses, and duration of therapy to reliably cure the majority of cases treated in Mongolia.

## Author Contributions

T-OB, KO, NB, GC, and YY conceived and designed the study. T-OB, KO, AB, TT, TM, and YY contributed by collecting samples. AE, BG, and DE provided *H. pylori* stool antigen test, microbiological, and histological assessment. T-OB, KO, and YY contributed to analysis and interpretation. T-OB, DD, TS, and GS enrolled and treated the patients and collected data. T-OB, KO, GC, NB, and YY drafted the manuscript. All authors read and approved the final manuscript.

## Conflict of Interest Statement

The authors declare that the research was conducted in the absence of any commercial or financial relationships that could be construed as a potential conflict of interest.

## Abbreviations

Clari-TT: clarithromycin-triple therapy
CI: confidence interval
ELISA: ånzyme linked immunosorbent assay
HpStAg test: *H. pylori* stool antigen test
ITT: intention-to-treat
M-BQT: modified bismuth quadruple therapy
MIC: minimum inhibitory concentrations
OR: odds ratio
PPI: proton pump inhibitor
PP: per-protocol
R: resistant
S: sensitive
SD: standard deviation
ST: sequential therapy
